# Biomarkers in Pulmonary Arterial Hypertension

**DOI:** 10.3390/diagnostics12123033

**Published:** 2022-12-03

**Authors:** Silvana Elena Hojda, Irina Camelia Chis, Simona Clichici

**Affiliations:** Department of Physiology, “Iuliu Hatieganu” University of Medicine and Pharmacy, Number 1–3, Clinicilor Street, RO-400023 Cluj-Napoca, Romania

**Keywords:** pulmonary arterial hypertension, biomarkers, right-sided heart failure

## Abstract

Pulmonary arterial hypertension (PAH) is a severe medical condition characterized by elevated pulmonary vascular resistance (PVR), right ventricular (RV) failure, and death in the absence of appropriate treatment. The progression and prognosis are strictly related to the etiology, biochemical parameters, and treatment response. The gold-standard test remains right-sided heart catheterization, but dynamic monitoring of systolic pressure in the pulmonary artery is performed using echocardiography. However, simple and easily accessible non-invasive assays are also required in order to monitor this pathology. In addition, research in this area is in continuous development. In recent years, more and more biomarkers have been studied and included in clinical guidelines. These biomarkers can be categorized based on their associations with inflammation, endothelial cell dysfunction, cardiac fibrosis, oxidative stress, and metabolic disorders. Moreover, biomarkers can be easily detected in blood and urine and correlated with disease severity, playing an important role in diagnosis, prognosis, and disease progression.

## 1. Introduction

There are five pulmonary hypertension (PH) groups, each of which is extensively described in the Guidelines for the Diagnosis and Treatment of Pulmonary Hypertension recently published by the European Society of Cardiology (ESC) [1]. Group 1 includes pulmonary arterial hypertension (PAH)—a severe pathology characterized by pulmonary vasoconstriction, vascular remodeling, and vascular media hypertrophy, followed by in situ thrombosis; it progresses to an increase in pulmonary artery pressure, the release of molecular mediators in plasma and, ultimately, right-sided heart failure (HF) and death [2,3]. Group 1 PAH comprises several subcategories, from idiopathic PAH (IPAH) and toxicity-induced PAH to other forms of inherited PAH (HPAP) associated with connective tissue diseases (CTD–PAH) or congenital heart diseases (CHD–PAH). The prognosis of PAH is based on the hemodynamic and biological parameters used to evaluate RV failure, as well as on treatment response. Early identification of the disease, along with the correct stratification of PAH, is essential for patients at high risk of major cardiovascular events. Thus, biomarkers are among the non-invasive, relatively accessible assays used in the diagnosis and staging of the disease. The progression of PAH is dynamically monitored with the help of a transthoracic ultrasound, while cardiac catheterization remains the gold-standard test, having both diagnostic and prognostic utility. For this reason, the existence of non-invasive assays for patient evaluation is essential. Therefore, clinical research in this regard has become increasingly exhaustive in recent years, so new biomarkers correlated with disease severity and progression are constantly emerging for better patient evaluation.

There are several maladaptive mechanisms involved in PAH, including endothelial dysfunction, inflammation, oxidative stress, cardiac fibrosis, pathological RV remodeling, cellular hypoxia, metabolic imbalance, and in situ thrombosis; these are essential mechanisms, each with one or more corresponding biomarkers [4]. In this article, we intend to detail most of the markers involved in precapillary PAH induction, focusing on each mechanism individually, as shown in Table 1. 

Table 1 presents biomarkers specific to different mechanisms involved in the onset of precapillary PAH.

## 2. Biomarkers Related to Heart Failure, Myocardial Stress and Injury, and Remodeling

**Natriuretic peptides** are molecules released by cardiac myocytes in response to increased heart pressure, volume overload [5], and wall stretching. Atrial natriuretic peptide (ANP) is released from storage granules, and its secretion is stimulated by atrial volume overload and atrial stretching. Brain (B-type) natriuretic peptide (BNP) is a 32-amino-acid polypeptide secreted by ventricular tissue in response to ventricular stretching of cardiomyocytes and is more stable than ANP [6]. ANP and BNP are released as prehormones and act at the renal level by stimulating diuresis and natriuresis, and by relaxing the smooth vascular musculature with veno- and arteriodilatory effects [7]. In recent years, attention has focused on the N-terminal fragment of BNP (NT-proBNP) as an alternative biomarker of BNP, which provides the same information and is still preferred in clinical practice as it has a longer half-life, greater stability, and higher assay accuracy compared to BNP [8]. BNP and NT-proBNP correlate with hemodynamic parameters and the New York Heart Association (NYHA) functional class, being independent predictors for the stratification of mortality risk [1]. They are released in response to myocardial ischemia, hypoxia, and ventricular wall stress. Both BNP and NT-proBNP are markers for screening, diagnosis, and prognosis, and they are also used to monitor the state of patients with acute and chronic heart failure [9]. Regarding PAH patients, clinical studies have shown that ANP levels change in response to pulmonary vasodilator therapy [10]. Nagaya et al. [11] were the first to show that BNP plasma levels have prognostic value in IPAH. Over time, it has been demonstrated that the natriuretic peptides BNP and NT-proBNP remain the only markers recommended by the latest guidelines of the European Society of Cardiology [1] for diagnostic algorithms, as well as for risk stratification, providing prognostic information [12,13], and playing a significant role in monitoring the efficacy of specific treatments [13,14]. They are routinely used in all centers specializing in pulmonary hypertension for monitoring RV myocardial stress and progression to RV failure. NT-proBNP is correlated with hemodynamic parameters obtained in cardiac catheterization, echocardiographic parameters of RV overload, and the 6-min walk test (6MWT) [12]. Over time, several cutoff values have been proposed for BNP and NT-proBNP. The latest recommendations of the REVEAL registry include stratification into four risk-assessment strategies [12,15] (a method also accepted by the ESC guidelines [1]): BNP < 50 ng/L and NT-proBNP < 300 ng/L for low risk (<5% mortality risk after 1 year), BNP 50–199 ng/L and NT-proBNP 300–649 ng/L for intermediate–low risk (5–10%), BNP 200–800 ng/L and NT-proBNP 650–1100 ng/L for intermediate–high risk (10–20%), and BNP > 800 ng/L and NT–proBNP >1100 ng/L for high risk (>20% mortality risk after 1 year). Moreover, in the DETECT study (Detection of Pulmonary Hypertension in Systemic Sclerosis), NT-proBNP was used to stratify the risk of PAH in patients with systemic sclerosis [16], and it was also found to be correlated with hemodynamic parameters.

**Serum cardiac troponins (cTn)** are regulatory proteins of thin actin filaments of the cardiac muscle [12]. The disruption of the myocyte membrane determines their release into the bloodstream, and they can be detected with high sensitivity [17]. cTnI and cTnT are the main biomarkers used in the diagnosis and prognosis of acute myocardial infarction [18]. The development of high-sensitivity cTn assays has further increased the accuracy of detection in various chronic pathologies, such as ischemic heart disease, left-sided heart failure (HF), and renal failure; cTn can therefore be interpreted as an increased risk marker for morbidity and mortality [19]. Various studies have assessed two mechanisms by which cardiac troponins are released during RV failure and PAH: microcirculation impairment, and demand–perfusion mismatch. Clinical studies have highlighted a direct correlation between plasmatic troponin levels and increased pulmonary vascular resistance (PVR), lower RV ejection fraction, lower mixed venous oxygen saturation (mvSatO_2_), and shorter 6MWT [17,20,21]. The presence of both cTnI and cTnT is associated with an increased mortality risk among patients with PAH [17,22]. Elevated levels of cTnI indicate patients with more advanced disease, constituting an independent prognostic role [21]. cTnT shows increased values only among patients with a reserved prognosis [22]. For this reason, the ESC guidelines recommend that troponin levels be measured both at the time of diagnosis and at least once per year or every time there is a clinical aggravation [1]. Therefore, it is not a marker of early disease, and the limitations regarding the interpretation of troponin levels in PAH are influenced by their association with renal failure or left-sided heart failure.

**Protein ST2** is part of the toll interleukin 1 superfamily receptor and is found in two forms: the transmembrane ST2 ligand (ST2L) is expressed in inflammatory cells, cardiomyocytes, and the endothelium [23], along with the blood-soluble suppression of tumorigenicity from sST2. The ligand for ST2 is interleukin 33 (IL-33), and this paracrine system (IL-33/ST2) plays an antifibrotic protective role [24]. However, the sST2 protein prevents IL-33 from binding to ST2L, thereby disrupting this cardioprotective effect. Over time, increased sST2 plasma levels have proven to be associated with acute or chronic HF as well as cardiac pathological remodeling [25,26]. Consequently, sST2 may be considered as an additional biomarker for adverse outcomes in this category of patients [27]. Levels above 35 ng/mL in patients with HF are associated with a high risk of hospitalization and death at 1 year.

In PAH, elevated levels of sST2 are correlated with inflammation, fibrosis, and/or pathological RV remodeling [28]. Clinical studies published in the past few years have highlighted that sST2 levels are statistically significantly correlated with cardiac index, PVR, RV dysfunction, and higher mean pulmonary arterial pressure (PAP) [29]. In addition, they could reflect PAH severity with a sensitivity and specificity of 83.3% and 78.6%, respectively [30,31]. Therefore, sST2 can be considered an independent predictor of clinical worsening, it can be correlated with disease severity or therapeutic efficiency, and it plays a prognostic role, independent of age or renal function [4].

There are statistically significant correlations between sST2 and mean PAP, NT-proBNP, and 6MWT in patients with PAH or chronic thromboembolic pulmonary hypertension (CTEPH) [31,32]. Moreover, it has been shown that sST2 can be considered to be a marker for therapeutic response in CTEPH patients treated with balloon angioplasty [31]. As such, sST2 is a complex marker reflecting diseases of the pulmonary vascular system and heart. Increasing evidence suggests that sST2 is a candidate biomarker in the context of PAH [30], as well as for risk stratification in patients with RV failure due to PAH [29].

**Cystatin C (CysC)** is a highly sensitive endogenous marker of renal filtration, with an important role in myocardial remodeling, and is able to predict acute left HF and cardiovascular mortality [33]. Compared to BNP and NT-proBNP, CysC serum levels are independent of muscle mass, gender, and age [34]. Clinical studies have shown a significant correlation with RV ejection fraction in HF populations [35]. As for PAH, Fenster et al. [36] studied the correlation between RV failure and plasmatic cystatin C concentration in a small group of subjects with preserved renal function. CysC showed a statistically significant correlation with function, morphology, and RV systolic pressure, all of which supported its role as a potential biomarker of PAH. Furthermore, CysC predicts long-term mortality and clinical events in patients with PAH–CHD, and it may also contribute to clinical decision-making regarding treatment intensity [37] Because CysC is an indicator of glomerular filtration, there are currently no data related to the association of PAH with advanced renal failure. Further information is required in this regard.

Regarding **homocysteine** levels, there is little information in the specialized literature. Small studies have highlighted homocysteinemia among patients with PAH and CHD, but without a statistically significant correlation [38,39]. Total homocysteine levels in plasma may be an important factor in the pathogenesis of PAH [40], but large-scale clinical trials are necessary for homocysteine to be recommended as a diagnostic or prognostic marker.

## 3. Inflammation Markers

The inflammatory process is an important mechanism in PAH, induced by sympathetic hyperreactivity due to decreases in cardiac output in the RV. Moreover, it reflects the degree of pulmonary arterial remodeling. A variety of pro- and anti-inflammatory markers have been studied over time in PAH patients.

**C-reactive protein (CRP)** elevation is broadly established as a predictor of numerous cardiovascular diseases and different types of PAH [4]. In PAH, a direct correlation between CRP and NYHA class, 6MWT, and right atrial pressure has been highlighted [41]. In PAH–CHD, an increase of over 10 mg/mL has been associated with an increased risk of death, so CRP is a simple but powerful marker of mortality in CHD–PAH patients. It should therefore be integrated in risk stratification and routine evaluation of these patients [42]. In CTEPH patients, a decrease in CRP was observed 12 months after endarterectomy [41]. Accordingly, this anti-inflammatory marker could be used for prognosis as well as for guiding therapeutic response in PAH.

**Red blood cell distribution width (RDW)** is a constantly measured laboratory marker. Increased levels are associated with anisocytosis, underlinked to a non-specific inflammatory process [43]. Over time, it has been studied in patients with various cardiovascular diseases (e.g., coronary arterial disease [44], pulmonary embolism [45], HF [46]). As for PAH, RDW can be considered a prognostic marker in patients with IPAH, along with GDF-15, IL-6, creatinine, and NT-proBNP levels [47], since its plasma concentration is correlated with disease severity. Another study, which included 77 patients with PAH and CTEPH, revealed that RDW meets all of the criteria to be used as a potential prognostic biomarker, also showing a significant decrease after the escalation of targeted drugs for PAH and CTEPH [48]. Decreased RDW levels are associated with good treatment response and better prognosis, but further prospective studies are still necessary in order to better understand the value of RDW in precapillary PAH.

**Growth differentiation factor-15 (GDF-15)** is a stress-responsive murine transforming growth factor-β-related cytokine that is highly expressed in the adult liver. It has recently been described as a non-specific inflammatory marker for various cardiovascular pathologies, as well as an independent prognostic marker in patients with acute pulmonary embolism and chronic left-sided HF. It is secreted in response to oxidative stress, inflammation, hypoxia, telomere erosion, and oncogene activation [49]. Moreover, it has been shown to be elevated in the sera of patients with IPAH. Nickel el al. [50] demonstrated that GDF-15 was abundantly expressed in the plexiform lesions of the pulmonary vascular endothelial cells, involved in both the apoptosis and the proliferation of the vascular pulmonary endothelium. In a group of 76 patients with IPAH, it was shown that GDF-15 can be considered an independent predictive marker of survival [50]. GDF-15 levels were correlated with biological (i.e., creatinine, uric acid, and NT-proBNP levels), clinical (i.e., NYHA class), functional (i.e., lower 6MWT), and hemodynamic parameters (i.e., mean right atrial and pulmonary capillary wedge pressures). High levels of GDF-15 are associated with an increased risk of mortality, independent of age or NT-proBNP; it is therefore a promising prognostic marker whose prognostic value should be detailed in future studies. Increased values have also been identified in the sera of patients with SSC–PAH, with GDF-15 levels positively correlated with PVR and plasma NT-proBNP levels [51].

**Galectin-3 (GAL3)**, a beta-galactoside-binding lectin, is a mediator for inflammation and fibrosis that is expressed in macrophages, neutrophils, eosinophils, and endothelial cells in response to tissue damage [4]; its involvement in cardiac remodeling and fibrosis is well known, and it plays a prognostic and diagnostic role in chronic HF [52,53]. GAL3 is approved by the American Heart Association as a marker for risk stratification in class IIb HF [54]. There are few studies focusing on the relationship between GAL3 and PAH; however, small-group studies have highlighted that RV failure in patients with PAH is statistically significantly correlated with functional and morphological RV changes [55] and high GAL3 concentrations [56]. The data in this regard are still scarce, and further studies are required.

**Cytokines** are considered to be prognostic inflammatory markers in numerous pathologies. Circulating levels of cytokines have been reported in PAH, with an important role in its progression. Soon et al. [57] showed increased levels of pro-inflammatory serum cytokines (e.g., interferon-gamma; interleukin (IL)-1beta, -2, -4, -5, -6, -8, -10, -12p70, and -13; tumor necrosis factor-alpha (TNF–α)) in patients with IPAH and HPAH compared to the control group. Moreover, in the same study, IL-6, -8, -10, and -12p70 were prognostic markers associated with low survival rates in IPAH and HPAH [57]. This information is supported by another study that showed increased levels of IL-6, vascular endothelial growth factor (VEGF), and platelet-derived growth factor (PDGF)-BB in patients with PAH [58].

**Osteopontin (OPN**) is a pleiotropic cytokine that, over time, has proven to be potentially related to mortality in PAH [59]. OPN levels were found to be correlated with age, 6MWT, NYHA class, mean right atrial pressure, and NT-proBNP in a study published by Lorenzen et al. in 2011 on patients with IPAH [59]. The data were also confirmed by Rosenberg et al. [60], who demonstrated that OPN is an independent prognostic marker of RV failure and pathological remodeling, most likely through its autocrine effect. Circulating OPN could be useful as a prognostic marker, in monitoring therapeutic response, and for improving risk stratification. Further studies on larger groups still need to be validated for use in clinical guidelines. Akin et al. emphasized another prognostic inflammatory marker, serum B-cell lymphoma 2 (sBCL2), found in high concentrations in children with PAH [61].

There is not as much information on the **neutrophil-to-lymphocyte ratio (NLR)** thus far. The first data related to the association between PAH and NLR appeared as early as 2013 [62]. Another study from that time highlighted that NLR could be correlated with the NYHA functional class and BNP in a small group of patients with PAH [63]. NLR ≥ 2.62 G/µL was associated with reduced five-year survival rates in PAH patients, with 69% sensitivity and 56% specificity [64]. Another recent study noted that high-NLR patients had lower 5-year transplant-free survival compared to the control group. Thus, NLR may be considered to be an independent predictor of survival, especially in women with PAH [65]. This simple marker could have prognostic value in PAH, but further studies are required in this direction.

**Macrophage migration inhibitory factor (MIF) and its receptor CD74** are overexpressed in muscular pulmonary arterioles of patients with IPAH and contribute to the abnormal pro-inflammatory phenotype [66]. Treatment with the MIF antagonist ISO-1 or anti-CD74 neutralizing antibodies reduced inflammatory cell infiltration and, in addition, reversed the development of pulmonary hypertension in rats [66]. In patients with PAH secondary to systemic sclerosis, high concentrations of MIF have also been found [67]; therefore, in the future, MIF could be considered as a possible prognostic marker for this pathology.

**Neopterin (NP)** belongs to the pteridines class; it is an inflammatory marker released by dendritic cells and macrophages that interacts with reactive oxygen species (ROS) in response to oxidative stress [68]. Over time, increased values of NP have been highlighted in various cardiovascular diseases, including HF and coronary heart disease. NP is considered to be a prognostic marker for these pathologies [69]. Thus far, the data related to the involvement of NP in PAH are very limited, but it seems to amplify PAH through its effects on ROS. The plasma concentrations of NP were elevated in patients with PAH and inoperable CTEPH, which are associated with various clinical outcomes.

**Adrenomedullin (ADM)** is a potent hypotensive and vasorelaxant peptide that can reduce blood pressure and PVR and increase pulmonary blood flow [70]. The concentration of ADM increases in direct proportion to the severity of PAH, and circulating ADM is metabolized in the lungs. These data suggest that ADM plays an important role in pulmonary vascular tone [71]. A recent study showed that intravenous administration of ADM reduced precapillary pulmonary hypertension by decreasing plasma aldosterone concentrations [72]. As a result, ADM could be considered to be a promising endogenic peptide in PAH treatment, as well as a vasoprotective factor [71].

## 4. Endothelial Cell Dysfunction and Pulmonary Arterial Smooth Muscle Cell (PASMC) Proliferation

**Asymmetric dimethylarginine (ADMA)** is a natural amino acid and an endogenous competitive inhibitor of nitric oxide (NO) synthase. Endothelial injury can increase its plasma concentration, resulting in decreased NO production, creating an inhibition of the NO/CGMP pathway with an increase in vascular tone. Reduced bioavailability of NO underlies the pathogenesis of pulmonary hypertension [73]. Over the years, clinical studies have highlighted the presence of ADMA in various cardiovascular pathologies, including myocardial infarction [74], CTEPH, and IPAH [73]. Several reports have shown high ADMA values in patients with IPAH, correlated with unfavorable pulmonary hemodynamics (e.g., PVR and CI) [75], but also in studies on patients with CTEPH [73] or CHD–PAH [38]. Increased serum ADMA levels could be used as an important prognostic marker, and in the mortality risk stratification and severity assessment of PAH.

**Circulating angiogenic modulatory factors:** Vascular endothelial growth factor (VEGF) signaling is involved in vascular remodeling and, implicitly, in the pathogenesis of PAH [76]. Plasma levels are increased in patients with IPAH [77], and the VEGF receptor 2 (VEGFR2) is overexpressed in plexiform vascular lesions. Soluble vascular endothelial growth factor (VEGF) receptor 1 (sVEGFR1) was also studied for its role in PAH. The soluble form of VEGF receptor 1 (also called soluble FMS-like tyrosine kinase 1 (sFlt-1)) was statistically significantly increased in patients with IPAH and CHD–PAH, being associated with the NYHA functional class [78]. The combination of sFlt-1 and PIGF has 83.7% sensitivity and 100% specificity for PAH [79]. In recent years, there has been an increased interest in connective tissue diseases (CTDs)—especially in the association between systemic sclerosis (SSc) and PAH. The potential role of these angiogenic circulating and inflammatory biomarkers in SSc screening is supported by numerous clinical studies, which have shown high levels of soluble vascular endothelial growth factor (VEGF) receptor 1 (sVEGFR1) [78,79] in patients predisposed to the development of PAH [80]. A significant decrease under prostanoid therapy has also been described and is considered to be a biomarker of treatment response [81]. Kylhammar et al. [3] also highlighted that the plasma levels of sVEGFR1 were correlated with both disease progression and worse outcomes. The value of other angiogenic and inflammatory biomarkers, such as placental growth factor (PlGF) [80], VEGF-A, IL-6 [58], IL-12 [80], and tumor necrosis factor-α (TNF-α) [58], seemed to best discriminate SSc patients who were on the verge of developing PAH.

**Aldosterone** is a mineralocorticoid hormone synthesized in the zona glomerulosa of the adrenal gland in response to a decrease in circulating blood volume [81]. Its role in vascular remodeling and fibrosis is well known, with several mechanisms through which it determines its effects, including activation of pathways that decrease NO levels, stimulation of inflammation and cell proliferation, extracellular matrix remodeling, and fibrosis [55]. Its role in vascular remodeling in PAH has been studied both in experimental models [82] and in subgroups of patients, where it was shown that the remodeling of the pulmonary arterioles increases vascular tone, causing irreversible right-sided HF (cor pulmonale). Perivascular fibrosis is reduced through pharmacological inhibition of aldosterone, along with the improvement of cardiopulmonary hemodynamics. Thus, aldosterone can be considered a partially modifiable marker in pulmonary circulation-right ventricle dysfunction [83]. Other studies consider galectin-3 and aldosterone to be potential tandem biomarkers of idiopathic PAH (IPAH) or PAH associated with PAH–CTD [55].

**Endothelin 1 (ET-1)** is considered to be a potent vasoconstrictor that stimulates the proliferation and migration of pulmonary artery smooth muscle cells (PASMCs). In the clinical studies published in previous years, statistically significant correlations have been highlighted between increased levels of ET-1 and hemodynamic parameters (e.g., mean PAP, CI, and disease severity) [84]. At the same time, ET-1 could be considered to be an ideal prognostic marker for disease progression, its levels correlating with responses to PAH-specific treatments [85]. According to the latest guidelines, the beneficial effects of endothelin receptor antagonists are well-known in PAH treatment [1]. In contrast, in recent years, it has been shown that COOH-terminal proendothelin 1 (CT-proET-1), a more stable form of ET-1, can provide superior prognostic information regarding ET-1 and death prediction at 12 months [86].

According to studies published in previous years, the **angiopoietin system (ANG**), consisting of angiopoietin 1 (ANG1) and angiopoietin 2 (ANG2) antagonists, could be involved in both disease staging and treatment response in patients with IPAH [87]. Kumpers et al. showed that ANG2 was statistically significantly correlated with CI, PVR, and mvSatO_2_, confirming its involvement in the pathogenesis of IPAH [88]. However, additional information has emerged more recently, suggesting that ANG2 is not associated with treatment response in patients with IPAH [89], although it may be used as a diagnostic and prognostic marker in Group 3 patients with PH.

**MicroRNAs (miRNAs)** are small non-protein-coding genes that function in RNA’s post-transcriptional regulation of gene expression. A new opportunity to sensitively detect changes in gene expression is available through RNA sequencing analysis (RNA-Seq) [90]. MicroRNA expression is associated with the progression of different vascular pathologies [40,91]. RNA-Seq was used in PAH patients to evidence changes in the transcriptomes of endothelial cells cultured from lung tissue and blood. Sarrion et al. noted that miR23a expression was correlated with pulmonary function parameters, including patient age, 6MWT, CI, and PVR. Overexpression of miR27a in patients with HPAH was correlated with 6MWT and was also associated with bone morphogenetic protein type 2 receptor (BMPR2) involvement in cell proliferation. The miR199a was correlated with 6MWT and mean PAP, while miR744 was correlated with PVR [91], and miR204 levels were lower in IPAH patients, supporting its importance in the pathogenesis of IPAH [92]. Another important miRNA associated with HPAH and IPAH was miR145, with a role in the pathophysiology of PAH [93]. The miR328 induces apoptosis in smooth muscle cells, inhibits IGFR1, and acts as a protective agent in PAH [94]. A novel relationship between BMPR2 dysfunction and reduced expression of collagen IV and ephrinA1 underlies the vulnerability to injury in PAH [90]. A recent study published by Rodor et al. in *Cardiovascular Research* identified a promising new candidate to target endothelial dysfunction in PAH—specifically, CD74, which is involved in the regulation of endothelial cell (EC) proliferation in vitro and may contribute to the progression of PAH, being considered a new candidate for future therapeutic development [95].

## 5. Hypoxia/Organ and Tissue Damage

Due to decreased cardiac output and peripheral hypoperfusion, biomarkers involved in the pathogenesis of PAH have been described with both diagnostic and prognostic roles.

**PaCO_2_**: Low partial pressure of blood carbon dioxide (PaCO_2_) in the arterial blood of patients with PAH is usually constantly low [96,97]. According to Hoeper et al., there is a prognostic correlation between the decrease in PaCO_2_ and the risk of morbidity and mortality, a correlation that is not reported for PO_2_, as this has no prognostic significance. The improvement of PaCO_2_ after three months of PAH-specific treatment was associated with increased survival rates [96]. PaCO_2_ can be considered to be an independent non-invasive prognostic marker, which is why PaCO_2_ measurement during follow-up in patients with PAH has independent prognostic value and can also be used to assess the risk of major cardiovascular events [97].

**Uric acid (UA)** is the final product of purine metabolism and may be increased under conditions of impaired oxidative metabolism and hemodynamic changes. Increased UA levels are dependent on age and sex but are found in various pathologies, such as cardiac overproduction, renal failure, use of diuretics, or left- or right-sided HF [98]. Various studies have shown that PAH patients with increased levels of UA are correlated positively with PVR and show increased mortality rates [99]. In a recent study, Wang et al. showed a significantly reduced survival rate in CTD–PAH with elevated blood UA values [100]. Interestingly, treatment with prostanoids showed much lower UA levels in pediatric patients [101]. The only limitation is that this marker is not specific for PAH, but correlated with other markers, which may have important prognostic significance.

**Copeptin** is a vasopressin precursor, a key regulator of body fluid homeostasis. Copeptin serves as a surrogate for plasma vasopressin levels [102] and may provide additional NT-proBNP information in the diagnosis and prognosis of patients with chronic HF [9,103]. In PAH, copeptin had a statistically significant correlation with NYHA class, 6MWT, and renal function, and its plasma levels were significantly lower after initiating PAH-specific therapy; it was also associated with shorter survival (*p*  <  0.001) in a study published in 2013 [102]. The best copeptin cutoff level was ≥24.2 ng/mL with a sensitivity of 90% and a specificity of 80% in 25 children with PAH–CHD [104]. This value was correlated with PVR, with high plasma levels being present in severe RV failure. In this case, copeptin is a clear prognostic marker. Therefore, it can be said that copeptin highlights neurohormonal imbalance due to the alteration of the RV function [4]. Consequently, high plasma levels of copeptin may provide significant independent prognostic information and could be used for risk stratification in PAH and other cardiovascular pathologies, where further extensive interventions are needed.

Any increase in central venous pressure is associated with aggravation of renal function and onset of prerenal kidney failure, an important prognostic marker. Significant kidney failure manifested by increased plasma **creatinine** values is common in PAH. An acute decline in renal function is a significant marker for in-hospital death and short-term mortality in PAH [105]. In addition to renal function, liver function is also very important in PAH. Plasma **bilirubin** levels higher than 1.2 mg/dL are associated with high mortality, hypoperfusion, and liver failure, with a possible prognostic role in PAH [106].

## 6. Metabolic Biomarkers

**Ghrelin**, an acylated 28-amino-acid peptide hormone, is the endogen ligand for the growth hormone secretagogue receptor [107]; it is produced by the gastrointestinal tract, especially by the stomach, and plays a role in regulating appetite [108]; it is also involved in various metabolic processes, in regulating body weight, and apparently has beneficial implications in cardiovascular diseases. In HF in rats, the administration of ghrelin was positively correlated with NYHA functional class [109]. Moreover, an experimental model showed that peripheral ghrelin administration may attenuate cardiac remodeling after MI [110]. Regarding PH, ghrelin-treated animals with hypoxia-induced pulmonary arterial hypertension showed significant improvements in PVR and RV hypertrophy [111]. Yang et al. highlighted that increased serum ghrelin levels were positively correlated with RV diameter, PVR, NT-proBNP, and NO concentration in IPH patients [112]. Since ghrelin is an endogenous hormone, it may represent a promising new treatment for PAH, as well as for other cardiovascular diseases.

**Fischer’s ratio (branched-chain amino acids/aromatic amino acids) and circulating amino acid profile (aminogram):** Over time, clinical studies have shown that the plasma concentrations of amino acids are significantly increased in patients with PAH, and the Fischer index was negatively correlated with NYHA, NT-proBNP, PVR, and 6MWT in a group of 160 patients with PH [113]. Thus, the plasma aminogram pattern and Fischer’s ratio are not elucidated in patients with PAH. Both could be considered to be prognostic markers of disease severity, but one major limitation is that large-scale investigations are still needed [40].

The impact of **high-density lipoprotein cholesterol (HDL-c)** concentration on cardiovascular pathology has been long studied from both experimental and observational perspectives. The inverse relationship between HDL-c and cardiovascular disease was described as early as 1964 in the Framingham Heart Study [114], and then, it was supported by animal studies and prospective studies with different populations [115,116]. Despite the fact that low plasma levels of HDL-c were associated with an increased risk of major cardiovascular events, elevated HDL-c has not demonstrated clear protection against atherosclerosis [116]. Regarding PAH, recent studies have highlighted that HDL-c exerts protective effects on the pulmonary vascular endothelium through several possible mechanisms, including vasodilatory activity, stimulation of NO production, anti-inflammatory properties, antioxidative properties, cytoprotection, modulation of glucose metabolism, regulation of glucose metabolism, and gene expression [116]. Heresi et al. showed that higher HDL-c levels in PAH patients were associated with slower disease progression [117], a finding also supported by Larsen et al., who concluded that higher HDL levels were associated with significantly lower mortality [118]. In addition, low plasma HDL-c levels are associated with higher mortality and clinical worsening in PAH patients [117]. Zhao et al. highlighted significantly lower values of HDL-c in patients with IPAH compared to a control group, directly proportional to the severity of the disease, i.e., short 6MWT, lower CI, and higher PVR [119]. Therefore, HDL-c can be considered to be an indicator of disease severity and an independent prognostic marker of long-term survival [118,119]. The plethora of data published thus far indicate the protective properties and survival benefits of HDL-c in PAH. Nevertheless, the clear mechanisms by which the lipid fractions exert their effects on pulmonary circulation are still under investigation. Studies on limited numbers of patients have shown low values of low-density lipoprotein cholesterol (LDL-c) and total cholesterol in IPAH and CHD-PAH, but without association with disease severity and heart failure [120].

Elevation in the triglycerides-high-density lipoprotein cholesterol **(TG/HDL-c)** ratio can be used as a marker of adiposity and an independent prognostic marker in patients with cardiovascular pathology; it has been shown to be correlated with abdominal obesity and higher body weight in healthy adults [121], but also with insulin resistance in some populations [122]. Studies have shown that there is an important correlation between TG/HDL-C ratio, in-hospital mortality, and major adverse cardiac events (MACEs) [123,124]. Patients with PAH usually have a low body mass index (BMI), but recent studies have highlighted elevated levels of circulating lipoproteins in such cases [125]. Increased TG/HDL-c ratios and low values of LDL-c are independent of BMI [126,127]. The mechanism that determines the increase in the TG/HDL-c ratio in PAH has not been fully elucidated. There are multiple inflammatory cytokines (e.g., IL-1β, IL-6, TNF-α) involved in increasing TG plasma levels and decreasing HDL-c concentrations [125]. A TG/HDL-c ratio > 3 can be considered to be a marker of systemic inflammation in IPAH [125] and a promising marker of severity progression in PAH [128]. Data are still limited regarding the role of lipid ratios in PAH. Future studies are required in order to evaluate the benefits of statin therapy in this patient category.

## 7. Oxidative Stress Biomarkers

**F2-isoprostane:** Over time, more and more PAH biomarkers have been highlighted in blood, but very few studies have evaluated their presence in urine. One of these, led by Cracowski et al., studied a urinary component, F2-isoprostane, a biomarker of lipid peroxidation, in 110 adults with PAH [129]. They found that F2-soprostane was an independent prognostic marker in PAH, as the only urinary component that was associated with an increased risk of death. They also suggested that the measurement of this urinary component in the urine of asymptomatic children with a family history of IPAH could be used for the early detection of disease progression [129]. However, the information obtained is supported by few clinical data; therefore, further clinical studies are still needed.

**Other oxidative-stress-related biomarkers:** Vasoconstriction induced by oxidative stress and decreased bioavailability are among the most important factors involved in PAH, increasing lipid peroxidation and reducing antioxidant defenses. In 2013, Reis et al. [130] published a study in which oxidative stress was evaluated based on plasma activity of reduced glutathione (**GSH)** and lipid peroxidation was expressed by malondialdehyde (**MDA)**. Thus, a reduction in GSH and vitamin E levels was highlighted, along with lipid peroxidation and a decrease in antioxidant defenses [130]. Therefore, it can be extrapolated that inflammation and oxidative stress may be considered to be important elements in PAH, but further investigations of oxidative stress markers in patients with pulmonary hypertension are required.

## 8. In Situ Thrombosis

In situ thrombosis and platelet aggregation have always been considered to be essential elements in the onset of plexiform lesions in PAH. **Von Willebrand factor (vWF)** is a large glycoprotein synthesized in the endothelium that plays an essential role in platelet aggregation and adhesion at sites of vascular injury; its elevated plasma levels have been used as markers of endothelial cell injury in an increasing number of pathologies [12]. Elevated plasma levels of vWF and its antigen (vWF:Ag) are associated with short-term prognosis in patients with pulmonary hypertension [131]. Another study highlighted that increased vWF levels were associated with a lower survival rate in patients with PAH [132]. In addition, increased levels of procoagulant microparticles have also been noted in PAH, associated with endothelial failure and thrombosis activation [133]. Therefore, circulating markers of endothelial damage, pro-inflammatory markers, and circulating microparticles could be essential tools in determining the severity of PAH [134]. **D-dimer**, measured by using the ELISA method, is a marker of cross-linked fibrin that indicates microvascular thrombosis. In patients with IPAH, increased D-dimer values were correlated with disease severity (i.e., NYHA, PVR, and mvSatO_2_) compared to the control group [135]. Nevertheless, these results need to be validated through extensive clinical trials.

The use of more biomarkers could improve risk assessment. A clinical study published in 2017 showed that a panel of nine different proteins, including several physiopathological mechanisms related to PAH, could be much more useful for patient staging, treatment response assessment, and prognosis compared to the existing clinical tools [136]. These are represented by ST2/IL1R1, the metalloproteinases TIMP1 and TIMP2, plasminogen, ApoE, erythropoietin (EPO), complement factors H and D, and insulin-like growth-factor-binding protein 1 (IGFBP1). However, further investigations and extensive population studies are required for this panel to be included in the current guidelines.

Moreover, we present the applicability of these biomarkers in clinical practice, along with their limitations, as shown in Table 2, together with potential biomarkers requiring further studies.

Because of space limitations, not all studies could be cited. Abbreviations: ADM, adrenomedullin; ADMA, asymmetric dimethylarginine; ANG, angiopoietin; BNP, brain natriuretic peptide; CRP, C-reactive protein; CysC, cystatin C; ET-1, endothelin 1; GDF-15, growth differentiation factor 15; HDL-c, high-density lipoprotein cholesterol; IL, interleukin; MIF, macrophage migration inhibitory factor; NLR, neutrophil-to-lymphocyte ratio; NT-proBNP, N-terminal fragment of pro-brain natriuretic peptide; OPN, osteopontin; PaCO_2_, partial pressure of blood carbon dioxide; PIGF, placental growth factor; PLC, platelet count; RDW, red blood cell distribution width; sFlt1, soluble FMS-like tyrosine kinase 1; TG/HDL-c, triglyceride–high-density lipoprotein cholesterol ratio; TnI, troponin I; TnT, troponin T; UA, uric acid; VEGF, vascular endothelial growth factor; vWF, von Willebrand factor.

## 9. Age and Sex Differences

Although PAH can appear at any age, it is most prevalent in young adults. Sex differences are observed in clinical practice in most PAH cases [138]. The European COMPERA registry shows a higher prevalence in women compared to men, at a ratio of 2.3:1 for the age group up to 65 years old, compared to 1.2:1 in the case of patients over 65 years old [139]. The equalization of this female-to-male ratio is influenced by the hormonal changes that occur during menopause [140]. The US National Institutes of Health (NIH) Registry [140] shows a 1.7:1 female-to-male ratio in the prevalence of PAH, while the REVEAL registry demonstrates a 4.1:1 ratio [141,142]. Moreover, hemodynamic profile differences between the sexes were reported in the REVEAL registry, including higher PAP/PVR and lower cardiac index in males compared to females [142]. The impact of sex hormones on pulmonary vasculature is complex and is still under investigation. Estrogens and androgens are pulmonary vasoactive components with variable effects on apoptosis and cell proliferation. It appears that sex hormones, especially estrogen, are the essential elements in understanding these mechanisms [138]. The literature describes an “estrogen paradox”. This condition has an increased prevalence in the case of women, and estrogen has been associated with a higher risk of PAH development. However, paradoxically, once affected, women have a better prognosis, improved long-term survival, and increased favorable responses to treatment compared to men [1,138,142]. The results obtained on experimental models have been contradictory over time; nevertheless, the majority have demonstrated a protective effect of estrogen on pulmonary vascularization. Females have less vascular remodeling and hemodynamic compromise than males and, in addition, older females develop fewer severe pulmonary vascular changes [143]. Moreover, testosterone is associated with maladaptive RV hypertrophy, greater RV volumes, and fibrosis in murines [144]. Dysregulation of estrogen synthesis and metabolism plays a major role in these sex-related differences [145]. Based on these data, there are some ongoing clinical trials to evaluate the effects of anti-estrogenic therapy in PAH (e.g., NCT03229499, NCT03528902), whose results are highly anticipated [138]. Indeed, further studies should be encouraged for a better understanding of the influence of sex on this disease, in order to establish new targeted treatments.

## 10. Immune System Cells

Cellular biomarkers can provide essential information about vascular endothelial function. These markers include circulating microvesicles and progenitor cells (PCs) [146]. These **endothelial microvesicles** (EMVs) are <0.1 μm in size. They are released by the endothelium in response to damage or apoptosis [146]. Circulating EMVs can be identified and quantified in peripheral blood vessels through flow cytometry [147]. Healthy individuals show only small amounts of EMVs; however, in PAH, increased levels of EMVs may be correlated with disease severity [146], especially in IPAH. It is therefore suggested that EMVs might play a role in the pathogenesis of PAH [146]. **Progenitor cells** are small immature cells produced in the bone marrow and released into circulation to replace damaged endothelial cells [148]. A reduced number of PCs in plasma circulation suggests decreased repair capacity of the vascular endothelium [148]. The EMVs/PCs ratio can be considered to be a marker of vascular competence, with significantly increased values in PAH [146].

In all types of PAH, numerous immune-cell infiltrations are evident at the pulmonary level, which contributes to pulmonary vascular remodeling. Among these cell infiltrations, we can identify cytotoxic and helper T cells, B cells, monocytes, macrophages, neutrophils, natural killer (NK) cells, dendritic cells (DCs), and mast cells [149].

**Macrophages** are key cells involved in the inflammatory response, with an essential role in the pathogenesis of PAH and arteriole muscularization through inflammation, as well as in endothelial dysfunction through the secretion of VRGF. There are two types of macrophages: classically activated M1 pro-inflammatory macrophages, and alternatively activated M2 anti-inflammatory macrophages. Inflammation stimulates local macrophage proliferation and recruitment of monocytes which, in turn, differentiate into macrophages [150]. Vergadi et al. showed an early accumulation of macrophages, especially those of the activated M2 phenotype, in the lungs of mice in a murine model of hypoxia-induced PAH [151]. Fan et al. reported that M1 macrophages cause apoptosis in endothelial cells, while M2 macrophages stimulate the proliferation of smooth muscle cells in a rat model of monocrotaline–induced PAH [152]. Therefore, inhibition of the function, production, and recruitment of macrophages can improve PAH, according to data from the literature [149,151].

**Mast cells** contribute to angiogenesis by producing mast cell proteases, which cause perivascular cellular lesions in PAH patients. The increased levels of mast cell proteases such as chymase and tryptase in lung tissue are directly proportional to vascular pathological remodeling and PAH severity [149,153].

**Natural killer (NK) cells** are able to identify and eliminate infectious cells via the degranulation of perforin-containing granules and granzymes [150]. Recent studies have confirmed the role of NK cells and CD8+ cytotoxic T cells in vascular remodeling as essential components in the vascular plexus lesions of IPAH and HPAP patients. Impairment of NK cells in PAH suggests a substantive role for innate immunity in the pathobiology of the disease [154]. In addition, experimental studies have shown that rats deficient in T cells more frequently present PAH with a worse prognosis [155]

Regulatory **T lymphocytes** are divided into natural Treg lymphocytes (nTregs), which express CD25+/CD4+, and induced Tregs (iTregs). Treg cells are essential in maintaining immune system homeostasis and inhibiting the immune reactions of autoreactive T cells [150]. Regarding PAH, the mechanisms involved are continuously being researched. Some reports describe the protective role of regulatory T cells against endothelial dysfunction [156]. Treg cell deficiency has been noted in IPAH, especially in women, signaling their association with the pathogenesis and development of PAH [149,156].

**B lymphocytes** produce antibodies, explaining the presence of antinuclear autoantibodies for pulmonary cells and fibroblasts in PAH. These endothelial autoantibodies stimulate apoptosis at the vascular endothelium level and contribute to vascular remodeling, with direct involvement in the pathogenesis of PAH [157]. The role of autoimmune damage comes from increased levels of IgA autoantibodies and plasmablasts [157]. The research carried out by Huertes et al. was among the first to highlight local autoimmunity and lymphoid tissue accumulation around remodeled vessels in PAH [158]. B-cell activation and immune complex deposition, in which decreased activity of T cells is a factor, lead to pathological vascular remodeling [149]. Moreover, Breitling et al. presented the mast cell-B lymphocyte axis as being involved in the occurrence of PAH through its effects on pathological vascular remodeling [159]. Another study demonstrated that the activation of B lymphocytes is associated with pulmonary fibrosis in systemic sclerosis patients [160]. For this reason, a phase II clinical trial with anti-CD20 antibodies is ongoing

The role of **dendritic cells (DCs)** in PAH has not been fully elucidated. DCs are involved in the activation of naive T cells and are also related to endothelial cells, antibodies, and fibroblasts. Clinical data suggest that DCs can accumulate in the adventitial pulmonary arteries of IPAH patients. In addition, complex lesions of human pulmonary arteries have shown transmural DC infiltration. Phenotyping revealed an immature DC profile in human and experimental pulmonary hypertension [161].

Inflammatory cell analysis has been a constant challenge over time. However, due to the complexity of immune cell populations, flow cytometry has proven to be a sophisticated approach to identify and quantify several inflammatory and immune cells. This technique can characterize cells from human blood, lavage samples, urine samples, and tissue samples (e.g., the lungs and pulmonary arteries for PAH) [162,163]. For the first time, Marsh et al. described the presence and regulation of two cell types in IPAH lungs: γδT cells and pDCs. With the help of high-throughput flow cytometry, they revealed the relationships between multiple inflammatory cells, autoimmune inflammation, and IPAH [163]. Rose et al. used flow cytometric methods to quantify decreased β-adrenergic receptor density in circulating white blood cells (WBCs) in PAH patients. Moreover, endothelial-cell-derived microparticles in urine samples were directly correlated with right ventricular function in PAH [164].

The latest literature data suggest the use of mass cytometry immunophenotyping (CyTOF) instead to flow cytometry for immunological studies. Flow cytometry is limited by some parameters that can restrict its utility. The advent of mass cytometry (CyTOF) has enabled high-dimensional and unbiased examination of the immune system, allowing for the simultaneous investigation of a large number of parameters. This is important for the deep and clear investigation of immune responses when sample sizes are limited [165]. The applicability of CyTOF has been validated in neoplastic pathology, but it has also demonstrated significant utility in other pathologies. Therefore, in 2020, the first report on CyTOF of blood mononuclear cells in PAH was presented, highlighting significant alterations of the innate and adaptive immune surveillance in IPAH and HPAP [166].

Consequently, microparticles can provide biological information about cell injury and apoptosis, but their clinical use is technically challenging. The availability of flow cytometry and of mass cytometry immunophenotyping (CyTOF) can help in quantifying microparticles from blood, urine, and tissues, and offers the possibility of more easily assessing immune biomarkers of vascular remodeling in PAH [164]. The roles of the immune system and autoimmunity in PAH have been extensively studied in recent years, but further research is still required for a deeper perception of the pathogenesis of the disease, in order to develop new targeted therapeutics and prevention strategies.

## 11. Conclusions

This article details some potential biomarkers previously assessed through clinical trials in PAH. So far, however, none of them have exhibited such high sensitivity and specificity that they could be used alone in establishing the diagnosis or in assessing the prognosis of patients with precapillary PAH. Given the complexity of the disease, it is unlikely that one marker alone will suffice. The great limitation of this review is that most of these markers were assessed through small clinical trials on a small number of patients. Therefore, most of the information should be treated with caution. Larger studies with higher numbers of patients are required for these biomarkers to be validated for use in clinical guidelines.

## Figures and Tables

**Table 1:** Circulating Biomarkers in PAH.

Heart Failure, Myocardial Stress and Injury, Remodeling	Inflammation	Endothelial Cell Dysfunction and Smooth Muscle Cell Proliferation	Hypoxia/Organ and Tissue Damage	Metabolic Biomarkers	Oxidative Stress Biomarkers	In Situ Thrombosis
BNP NT-proBNP Troponins (T, I) Cystatin C sST2 Homocysteine	CRP Galectin-3 RDW GDF–15 Cytokines: (IL-6, IL-8, IL-10, IL-12p70) OPN Neopterin MIF NLR ADM	ET-1 CT-proET-1 ADMA ANG1 ANG2 Aldosterone sVEGFR1 miRNAs	PaCO_2_ Creatinine Uric acid Copeptin Bilirubin	HDL-c TG/HDL-c ratio Fischer’s ratio Ghrelin	F2-isoprostane Vitamin E Glutathione	vWF D–dimers

**Biomarkers in PAH:** ADM, adrenomedullin; ADMA, asymmetric dimethylarginine; ANG, angiopoietin; ANP, atrial natriuretic peptide; BNP, brain natriuretic peptide; CRP, C-reactive protein; CT-proET-1, COOH-terminal proendothelin 1; ET-1, endothelin 1; GDF-15, growth differentiation factor-15; HDL-c, high-density lipoprotein cholesterol; IL, interleukin; MIF, macrophage migration inhibitory factor; miRNAs, micro-RNA; NLR, neutrophil–to–lymphocyte ratio; NT-proBNP, N-terminal fragment of pro-brain natriuretic peptide; OPN, osteopontin; PaCO_2_, partial pressure of blood carbon dioxide; PLC, platelet count; RDW, red blood cell distribution width; TG/HDL-c, triglyceride–high-density lipoprotein cholesterol ratio; TnI, troponin I; TnT, troponin T; UA, uric acid; VEGF, vascular endothelial growth factor; vWF, von Willebrand factor.

**Table 2:** How to use biomarkers in the clinical management of PAH: an extended table showing different biomarkers discussed in this review.

	Biomarkers	Subjects	Important Findings
Disease progression	BNP/NT-proBNP	61 patients with IPAH [13] 1655 patients with PAH [15]	Correlation with hemodynamic parameters (i.e., PVR, CI, mean RAP) and exercise capacity [13,15]
	ET-1	16 patients with IPAH [84]	Positive correlation with PVR and disease severity [84]
	RDW	139 patients with IPAH [47]	Related to disease severity [47]
	OPN	71 patients with PAH [60]	An independent predictor of RV dilatation and dysfunction [60]
	HDL-c	76 IPAH patients [119]	Serum HDL cholesterol levels were significantly decreased in patients with IPAH—an indicator of disease severity and progression [119]
	ANG2	81 IPAH patients [87]	ANG2 was statistically correlated with CI, PVR, and mixed venous oxygen saturation (mvSatO_2_) [87]
	sFlt1, PIGF	62 patients with IPAH [78]	Early-stage diagnostic evaluation [78]
Therapeutic response	BNP/NT-proBNP	1142 GRIPHON patients [14]	First evidence of an association between NT-proBNP levels and treatment response [14]
	sVEGFR1	62 patients with IPAH [79]	Treatment response biomarker [79]
	ADM	13 patients [71]	Promising endogenous peptide for the treatment of pulmonary hypertension [71]
	PaCO_2_	101 patients with IPAH [96]	Increase in survival rate after treatment; limitations: retrospective design, all data came from a single center [96]
	CRP	1004 patients with PAH [41]	Predicting responses to therapy in PAH [41]
	Cystatin C	59 patients with CHD–PAH [37]	May be attributable to clinical decision-making regarding treatment intensity [37]
Prognosis	BNP/NP-proBNP	1426 patients with PAH [137] 1655 patients with PAH [15]	Strong predictor of 5-year survival [137] Four–stratum model risk stratification more prognostically relevant [15]
	TnT	56 patients with PAH [17]	Elevated levels represent more advanced disease [17]; does not represent a marker of early disease
	TnI	255 PAH patients [20]	Used in routine clinical practice; more severe hemodynamic and cardiac structural failure; high risk of mortality; poor outcomes; low specificity [20]
	IL-6, -8, -10, and -12p70	60 patients with IPAH and HPAH [57]	Prognostic factors associated with low survival rates in IPAH [57]
	PaCO_2_	204 patients with IPAH [97]	Independent prognostic value, risk assessment for major cardiovascular events [97]
	Renal function (i.e., creatinine, BUN, uric acid)	50 patients with CTD–PAH [100]	UA levels might predict the severity and clinical prognosis of the disease [100]
	Bilirubin	37 patients with PAH [106]	Risk factor for death in patients with PAH [106]
	Cystatin C	14 patients with PAH [36] 59 patients with CHD–PAH [37]	CysC was accurately correlated with cardiac and hematological dynamics, e.g., RV pressure, function, and morphology [36] CysC may represent a novel biomarker of PAH, predicting long-term mortality and clinical events in patients with CHD–PAH [37] Limitation: small sample sizes
	sST2	104 patients [29] 40 patients with IPAH [30]	sST2 was correlated with disease severity and was a significant predictor of clinical worsening in patients with PAH [29,30]
	RDW	139 patients with IPAH [47]	Potential prognostic biomarker related to disease severity, and may be used to predict survival [47]
	CRP	1004 patients with PAH [41] 225 CHD–PAH patients [42]	Predicting outcomes of PAH [41] Should be incorporated in risk stratification and routine assessment of CHD–PAH [42]
	GFR-15	76 patients with IPAH [50]	A new and promising prognostic biomarker of the risk of death in patients with IPAH [50] Deserves further investigation
	OPN	70 patients with IPAH [59]	Independent predictors of mortality [59]
	NLR	71 patients with PAH [63]	Poor overall 5–year survival in PAH patients [63]
	ADMA	57 patients with IPAH [75] 30 patients with CHD–PAH [38]	High serum ADMA concentrations were associated with unfavorable pulmonary hemodynamics (i.e., higher mPAP and PVR) and worse outcomes in patients with IPAH and CHD–PAH [38,75]
	Copeptin	25 children with CHD–PAH [104] 92 PAH patients [102]	Predicting poor outcomes [104] Circulating levels of copeptin were independent predictors of poor outcomes—a potentially useful biomarker in PAH [102]
	HDL-c	69 patients with PAH [117] 76 IPAH patients [119] 227 PAH patients [118]	Low plasma HDL-c was associated with higher mortality and clinical worsening in PAH [117] Lower HDL-c levels were associated with lower event-free survival [119] Higher HDL levels were associated with significantly lower mortality [118]
	TG/HDL-c ratio	122 patients with PAH [128]	TG/HDL-c is a promising independent risk factor for the severity of PAH [128]
	sVEGFR1	97 PAH patients [78]	Correlation with disease progression as well as worse outcomes [78]
	vWF	66 PAH patients [132]	High vWF levels at baseline and follow-up were associated with worse survival [132]
